# Validation and Optimization of an *Ex Vivo* Assay of Intestinal Mucosal Biopsies in Crohn’s Disease: Reflects Inflammation and Drug Effects

**DOI:** 10.1371/journal.pone.0155335

**Published:** 2016-05-12

**Authors:** Kasper Vadstrup, Elisabeth Douglas Galsgaard, Jens Gerwien, Marianne Kajbæk Vester-Andersen, Julie Steen Pedersen, Julie Rasmussen, Søren Neermark, Marianne Kiszka-Kanowitz, Teis Jensen, Flemming Bendtsen

**Affiliations:** 1 Gastro Unit, Medical Division, Hvidovre University Hospital, Hvidovre, Denmark; 2 Faculty of Health Sciences, The Panum Institute, University of Copenhagen, Copenhagen N, Denmark; 3 Biopharmaceutical Research Unit, Novo Nordisk A/S, Maaloev, Denmark; Instituto Nacional de Ciencias Medicas y Nutricion Salvador Zubiran, MEXICO

## Abstract

Crohn’s disease (CD) is a chronic illness demanding better therapeutics. The marketed biologics only benefit some patients or elicit diminishing effect over time. To complement the known methods in drug development and to obtain patient specific drug responses, we optimized and validated a known human explant method to test drug candidates and pathophysiological conditions in CD intestinal biopsies. Mucosal biopsies from 27 CD patients and 6 healthy individuals were collected to validate an explant assay test where the polarized tissue was cultured on a novel metal mesh disk, slightly immersed in medium imitating an air-liquid interphase. After culture in high oxygen for 24 hours with or without biological treatment in the medium, biopsy integrity and penetration of antibodies was measured by immunohistochemistry (IHC). Nine cytokines were quantified in the conditioned medium as a read-out for degree of inflammation in individual biopsies and used to evaluate treatment efficacy. The biopsies were well-preserved, showing few structural changes. IHC revealed tissue penetration of antibodies demonstrating ability to test therapeutic antibodies. The cytokine release to the medium showed that the assay can distinguish between inflammation states and then validate the known effect of two treatment biologics confirmed by a detection panel of five specific cytokines. Our data also suggest that the assay would be able to indicate which patients are responders to anti-TNF-α therapeutics, and which are non-responders. This study demonstrates this version of an *ex vivo* culture as a valid and robust assay to assess inflammation in mucosal biopsies and test of the efficacy of novel drug candidates and current treatments on individual patients–potentially for a personalized medicine approach.

## Introduction

Crohn’s disease (CD), an inflammatory bowel disease (IBD), is a complex immunologically mediated chronic illness that arises due to a dysregulated immune response to yet unidentified antigens in the gastrointestinal system. CD is characterized by transmural inflammation with a preference for colonic and ileal appearances[1, 2]. CD typically arises between 20 and 30 years of age with symptoms such as abdominal pain, fever, diarrhea, and weight loss[3]. Despite newer and better therapies, Crohn’s disease often presents a heavy everyday burden, sometimes leading to surgery and disability[4, 5]. The precise etiology of Crohn’s disease is still unknown, but evidence points towards genetic[6] and lifestyle factors such as diet and smoking being involved[7, 8]. The pathogenesis involves immune factors such as pro-inflammatory T cells producing cytokines involved in induction and perpetuation of the intestinal inflammation[9, 10]. Established older treatments, like steroids, aim at inhibiting the entire immune response, while newer biological treatments focus on specific immunological pathways, such as targeting of effector cytokines or cell migration. However, these new drugs lead to long-term remission in only a minority of CD patients, whilst others experience a diminishing effect over time. This leaves an unmet medical need for more effective therapeutics and better outcome predictions for patients[11, 12]. Personalized medicine offers a promising approach where risk stratification and efficacy concerns are taken into consideration. Unfortunately, only few assays are available and new, robust and validated tests are needed[13–15].

The use of animal models of colitis is the predominate method for preclinical development and testing of new biological therapies. While these models can be successfully used to demonstrate proof-of-principle, mechanism of action and safety of new drugs, they have limitations regarding mimicking disease, translation into humans, and uncertainty of effect in a clinical setting[16]. *In vitro* methods to initially test mode of action or efficacy of drugs normally comprise simple cell-cell co-culture assays or newer, more sophisticated, 3D cell models[17–19]. These models are also not readily translational into responses in human tissue and as in animal models, an inflammatory environment will have to be artificially induced[20, 21]. Other *ex vivo* assays with tissue from patients involve isolating mixed cell populations and incubating them in a dish, or submerging the full tissue in medium before analyzing the production of cytokines or transcription factors. Results from cell cultures are compromised by lost integrity of the microenvironment and the submerged biopsies are affected by the culturing method where tissue conservation is poor and cell death occurs[22, 23].

To meet the demand for a human-like, preclinical efficacy test and to deliver better personalized medicine testing of efficacy of drugs on individual patient material, a validated and robust *ex vivo* assay would be a major step forward. In the current study, we validate the method for the sensitive testing of drugs and prediction of responses in an *ex vivo* culture platform, using well-preserved biopsies from Crohn’s disease patients with a robust read-out. The model is based on a culturing system that has been used before in different settings and is designed to keep the tissue alive in an air-liquid interphase, similar to the natural environment[24–28], but not previously validated with regard to antibody penetration and reaction to biologics.

## Materials and Methods

### Study population

CD patients referred for endoscopy or surgery comprised the study population of 27 subjects. Inclusion criteria were age above 18 years, no pregnancy, HIV negative and without infections at time of endoscopy or surgery. Six healthy controls were recruited from a colon cancer screening program and revealed no symptoms or signs of intestinal disease at endoscopy. Subjects were scored beforehand by the Harvey Bradshaw index (HBI), an interview-based standard clinical tool for estimating disease state[29]. HBI scores ≤4 is considered as remission. The endoscopist scored the endoscopic findings in agreement with the simple endoscopy score (SES)[30]. Of the CD patients, five were determined by the endoscopist to be in remission showing no visible inflammation and were registered as uninflamed. Eight of the 27 patients underwent surgery and donated visibly inflamed resections. Blood and stool samples were collected from CD patients for biochemical analyses of C-reactive protein (CRP) and fecal calprotectin, respectively. All patients were recruited at Amager-Hvidovre Hospitals, Denmark, and signed written consents under the ethical protocol H-1-2012-137 approved by The Danish National Committee for Health Research Ethics.

Porcine intestinal samples were used for establishment and optimization of assay. All procedures involving animals were performed in accordance with internationally accepted principles for the care and use of laboratory animals.

### Ex vivo explant assay

**Fig 1:**
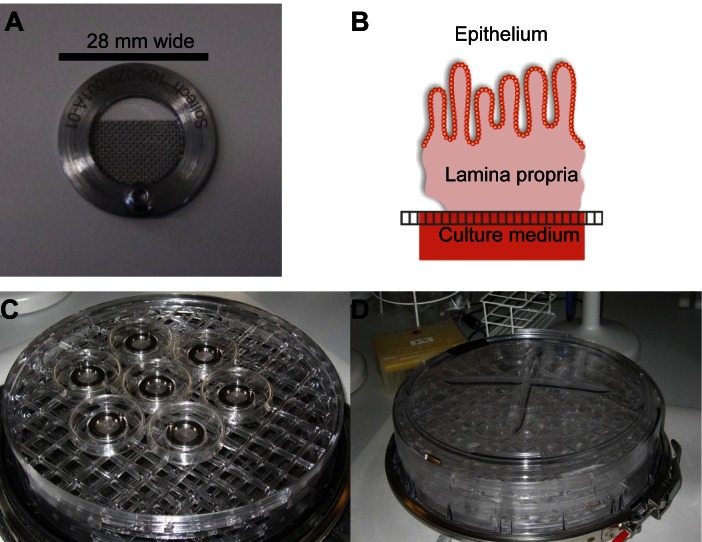
Explant assay setup. (A) Reusable T-disk with a central metal mesh designed to fit into the Falcon center-well organ culture dish. (B) Cartoon of a biopsy placed on the T-disk in air/liquid interphase oriented with the epithelial cell layer upwards and the cut surface downwards, slightly submerged in the medium. (C) Picture of the open incubator. (D) Picture of the sealed incubator.

Small intestinal and colonic biopsies and resections were transported on ice in Dulbecco's Modified Eagle Medium (DMEM) liquid (High Glucose) with GlutaMAX™ I (Gibco, Grand Island, NY) and processed within one hour. Biopsies from a specific inflamed or non-inflamed site were taken as closely together as possible to ensure low variability (<5 cm^2^). Building on the *ex vivo* organ culture model presented by Tsilingiri et al.[27] and originally based on Browning and Trier[24], we cultured the biopsies with comparable medium on specially constructed steel grids (dubbed T-disks) in an airtight chamber filled with a carbogen mixture of 95% O_2_ and 5% CO_2_ for 24 hours (Fig 1). Based on pilot experiments on both porcine and human tissue, the culture medium was composed of 1000 μl DMEM with GlutaMAX™ I supplemented with 10% fetal bovine serum, 1% non-essential amino acids, 1% Sodium Pyruvate, 1% Penicillin/Streptomycin (all Gibco), 50 mg/ml Gentamicin (Sigma-Aldrich, St. Louis, MO) and 10 μg/ml Insulin-Transferrin-Selenium-X (Sigma-Aldrich). The medium was dispensed into a center-well organ culture disk (Falcon, Corning, Tewksbury, MA). One ml medium was dispensed into the outer boundary of the culture disk to control humidity. Biopsies were carefully placed with an apical to basolateral polarity on the T-disk with the latter side in contact with the medium below. For surgical resection, the mucosal layer was stripped from the tissue by scalpel and biopsies were punched out with a 3 mm biopsy punch (Miltex, York, PA) and positioned in the same manner (only colon). The T-disk was compared to a 3.0 μm pore size transwell insert (Costar, Corning) or to submerging of the biopsy directly in medium. For validation, the following monoclonal antibodies were added to the culture medium (10 μg/ml): anti-human CD3 (clone OKT3, eBioscience, San Diego, CA) with IgG2a isotype (eBM2a, eBioscience), anti-human CD31 (WM59, eBioscience) with IgG1 isotype (Abcam, Cambridge, UK) and anti-human TNF-α (Infliximab, Janssen, Horsham, PA) with IgG1 isotype (humanized IgG1, Novo Nordisk, Maaloev, Denmark). Only colonic biopsies were used in validation to reduce potential bias. When testing with Infliximab, only patients not currently on biological medicines were included.

### Histology

Sections (3 μm thick) of paraformaldehyde-fixed and paraffin-embedded (PFPE) colonic mucosa biopsies were mounted onto Superfrost Plus slides (Fisher Scientific), dried for 1 hour at 60°C and stored at 4°C until use. Deparaffinised and rehydrated sections were stained with H&E (haematoxylin (Ampliqon, Skovlunde, Denmark) and eosin (Sigma-Aldrich, Brøndby, Denmark), dehydrated and mounted in Pertex (Histolab Products). Scanned images were recorded with a Nanozoomer (Hamamatsu, Japan) and samples for inflammation analysis were analyzed blinded at a three point scale of inflammation degree: non-inflamed, mildly/moderately inflamed, severely inflamed. Biopsies were scored in a blinded manner by two investigators.

#### Immunohistochemistry (IHC)

Resection tissue or biopsies were embedded in OCT compound (VWR, Leuven, Belgium) and snap frozen in 2-methylbutane cooled by dry ice. Sections of cryopreserved tissue blocks were cut (5 μm), air-dried overnight and used immediately, or stored desiccated in Kartel boxes at -80°C and brought to room temperature (RT) before use. Unless otherwise indicated, all steps were performed at RT. Sections were washed between each step in Tris-buffered saline (TBS) with 0.01% Tween20 (VWR). Sections were fixed in acetone (-20°C) for 10 minutes and air-dried for 30 minutes. Endogenous peroxidase activity was blocked by Dual Block (DAKO) for 5 minutes and endogenous biotin (Biotin Blocking System (Invitrogen)) according to manufacturer’s instructions. Unspecific binding was blocked by pre-incubation in TBS with 3% human serum (Jackson ImmunoResearch), 7% donkey serum (Jackson ImmunoResearch), 3% bovine serum albumin (BSA) fraction V (Hyclone) and 3% non-fat dry milk (DIFCO) for 2 hours. To detect tissue penetration of antibodies added to the culture medium, biotinylated anti-mouse IgG2a or IgG1 isotype-specific secondary antibodies (Jackson ImmunoResearch) were diluted to 0.17 μg/ml in TBS with 3% human serum, 7% donkey serum, 3% BSA and 0.5% non-fat dry milk and applied onto tissue sections for 1 hour. For comparison, CD3 positive cells in the biopsies were detected by direct IHC using a primary anti-human CD3 antibody (Rabbit SP7, Thermo Scientific, Waltham, MA) that was diluted to 0.17 μg/ml, incubated overnight at 4°C and detected with a biotin-tagged secondary antibody (donkey anti-rabbit, Jackson) at 0.17 μg/ml both in the same buffer as above. Antibody binding was detected by avidin-biotin-horse radish peroxidase complex (Vectastain, Vector) in TNB for 30 minutes followed by biotinylated tyramide (Tyramide signal amplification (TSA) kit, NEL700, Perkin Elmer) in amplification buffer for 6 minutes and Vectastain in TBS for 30 minutes. Sections were washed with TBS before and after TSA incubation. Antibody binding was visualized by diaminsobenzidine and nuclei were counterstained with hematoxylin. Sections were mounted in Pertex (Histolab Products) and examined in an Olympus AX70 microscope before photographed as above.

### Cytokine quantification

All cytokine measurements were done with Bio-plex Pro technology on a Bio-plex 200 multiplex system (Biorad, Hercules, CA), detecting the cytokines interleukin (IL)-1β, IL-2, IL-10, IL-12p70, IL-13, IL-17A, GM-CSF, IFN-γ and TNF-α as per Biorad instructions. When a measurement failed to be above detection level defined by the standard curve, the lowest value in the data set served as a replacement instead of zero.

### RNA quantification and quality analysis

Small intestinal biopsies were stored in RNA stabilization solution RNAlater® (Ambion, Life Technologies, Carlsbad, CA) post assay and for total RNA purification lysed in 400 μl of TRIzol (Invitrogen, Life Technologies, Carlsbad, CA). Chloroform (80 μl) was added and whirl mixed for 15 sec. Homogenates were incubated 2–3 min at RT and centrifuged for 15 min at 10,000g (4°C). The water phase containing the RNA was further purified using RNeasy MinElute Cleanup Kit (Qiagen, Venlo, Netherlands). RNA concentration measured on a nano-drop 2000 (Thermo Scientific) and the integrity was confirmed on an Agilent 2100 Bioanalyzer using total RNA nano chips (Agilent Technologies, Santa Clara, CA, USA). RNA analysis of explant biopsies from 8 CD patients were performed, grading the quality post assay to prove cell survival. The RNA Integrity Number (RIN) score of 0 through 10 (perfect quality) is the gold standard for evaluating the integrity of the RNA[31].

### Statistical analyses

Results were visualized with mean and standard deviation (SD) and analyzed with the Prism 5 professional software (Graphpad, San Diego, CA). All graphs are with a log10 y-axis. A paired t-test of the mean was used to compare a specific cytokine release, when analyzing biopsies coming from the same patient with the same inflammation degree. Two-way analysis of variance (ANOVA) was used to determine differences in results between treatments or disease states across the panel of the cytokine releases showed. The means of cytokine quantifications from more than one biopsy from the same patient were combined to a table of means for statistics between more patients. Cytokine data is normalized to weight of the biopsies and presented in pg/ml/100 mg of tissue. All relevant *P* values are shown in each figure. *P* < .05 was considered statistically significant.

All authors had access to the study data and have reviewed and approved the final manuscript.

## Results

### Characteristics of the patient population

Clinical characteristics of patients and normals are listed in Table 1. Five of the CD patients showed no visible inflammation and were determined by the endoscopist to be in remission (uninflamed). The CD patients were mostly women (56%) and median age was 32 years, whereas the healthy controls were mostly men (67%) with a median age of 49 years. Ten (45%) of the inflamed patients had colonic disease (L2, Montreal Classification), seven (32%) had ileocolonic disease (L3) and 5 only ileal disease (L1). The interview-based HBI score revealed no difference between the inflamed and uninflamed group. Serum CRP and fecal calprotectin both showed a trend towards higher concentrations in the inflamed population compared to uninflamed and healthy controls (CRP data only for the latter group), but the variability was considerable and no statistically significant differences were observed.

**Table 1:** Clinical features of patients and healthy controls.

Characterizations and parameters	Inflamed CD n = 22	Uninflamed CD n = 5	Healthy control n = 6
**Age (years), median (range)**	32 (18–63)	32 (22–46)	49 (31–72)
**Gender, number**			
Female	13	2	2
Male	9	3	4
**Donated tissue location, number (biopsies/surgical resections)**			
Ileum	7 (5/2)	0 (0/0)	0 (0/0)
Colon	15 (9/6)	5 (5/0)	6 (6/0)
**Disease phenotype, number (% of total)**			
Colonic (L2)*	10 (45%)		
Ileocolonic (L3)*	7 (32%)		
Ileal (L1)*	5 (23%)		
**Medication up to 3 months prior, % of total**			
5-ASA	0	0	0
Corticosteroids	10	0	0
Azathioprine/6-Mercaptopurine	24	60	0
Anti-TNF-α agents	5	40	0
Without medication	61	20	100
**SES, mean (range)**	12 (2–28)	1.2 (0–3)	0 (0)
**HB score, mean (range)**	6.4 (2–17)	4.4 (1–6)	0 (0)
**CRP (mg/l), mean (range)**	44.2 (0.3–190)	23.2 (1–83)	3.5 (0.3–12)
**Fecal calprotectin (mg/kg), mean (range)**	1719 (30–3600)	497 (30–971)	

5-ASA, 5-aminosalicylic acid; TNF, tumor necrosis factor; SES, Standard Endoscopic Index; HB, Harvey Bradshaw; CRP, C-Reactive Protein (normal < 10 mg/l); Fecal calprotectin (normal < 30 mg/kg).

*Montreal Classification.

### Development of the T-disk for optimal explant culture conditions

In a series of pilot studies, we used porcine intestinal biopsies to compare T-disk culturing with submerging in culture medium. Histopathology evaluation of the biopsies after 24 hours of culture showed that T-disks markedly improved preservation of tissue integrity and cell survival compared to submerged biopsies (S1 and S2 Figs for small intestine and colon, respectively). Compared to fresh, uncultured biopsies, we observed, however, characteristic morphological changes of the cultured biopsies, such as accumulation of debris at the luminal surface, loss of some mucus-containing goblet cells, and ischemia-induced sub-epithelial (Gruenhagen) spaces[32]. Pilot experiments, moreover, supported the updated method of Tsilingiri et al.[27] in terms of culture time, oxygen levels and culture media additives on tissue preservation. The only exception was the addition of epidermal growth factor, which we found not to have an impact on tissue survival when analyzed by histology. Importantly, intestinal biopsies from CD patients cultured on either T disk or completely submerged in medium showed similar histomorphology as porcine biopsies (S3 Fig).

**Fig 2:**
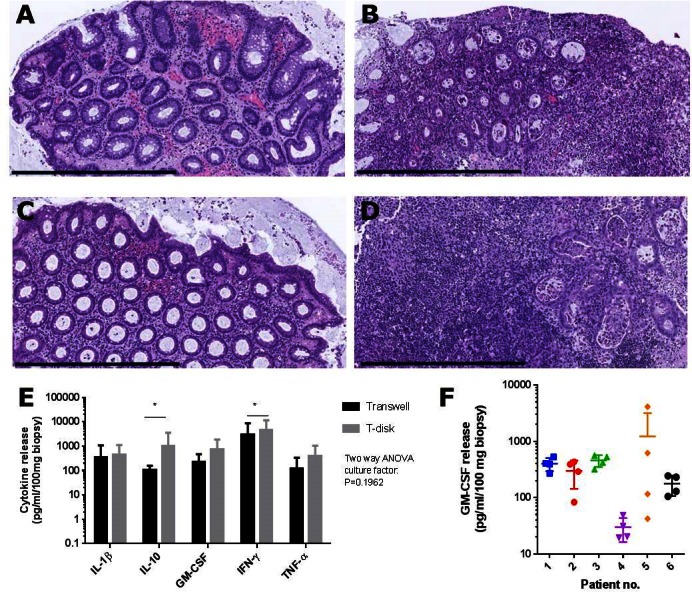
Preservation of tissue morphology and robust cytokine release in the explant assay. Non-inflamed (A, C) and inflamed (B,D) biopsies from the same patient were cultured on T-disks (A, B) or transwells (C,D) for 24 hours and processed for histology. Representative pictures of H&E-stained paraffin-sections (n = 5 patients). Bars = 600 μm. (E) Spontaneous release of the cytokines IL-1β, IL-10, GM-CSF, IFN-γ and TNF-α from inflamed biopsies into the culture medium measured by Bio-Plex. Biopsies from the same patients were cultured on either T-disk or transwell as indicated. No difference in cytokine release was observed when comparing T-disk and transwell for all 5 cytokines in combination (*P* > 0.05, two-way ANOVA), but when analyzed alone, IL-10 and IFN-γ were significantly increased with T-disk (*P < 0.05, t-test). 2 biopsies per patient (n = 6). (F) Spontaneous release of GM-CSF from 6 inflamed patients, 4 biopsies per patients taken as closely as possible from each site, all shown individually. Data show mean ± SEM.

Having demonstrated with both human and porcine biopsies that the T-disk is superior over the submerged culture condition, we next compared the T-disk with the transwell insert. The two different platforms, that both offer culturing in an air-liquid interface, performed equally well with regards to histomorphological preservation of the tissue (inflamed or non-inflamed, Fig 2). Variation from patient to patient was observed with regards to morphological changes as a result of *ex vivo* culturing, which may influence the cytokine read-out.

To assess the quality of RNA isolated from cultured biopsies, RIN score was measured for 24 biopsies from 8 inflamed CD patients. The mean RIN score was 9.479 ± 0.3788, showing low degree of RNA degradation[31]. Together, histomorphological and RNA analyses suggest that mucosal biopsies are well-preserved after *ex vivo* culture in the explant assay on T disk for up to 24 hours.

### 5 cytokines in the conditioned medium reflecting the level of inflammation in the cultured biopsies

Nine different cytokines were analyzed by multiplex quantification. Levels of IL-2, IL-12p70, IL-13 and IL-17a often fell below detection level, making these cytokines unreliable as biomarkers of inflammation. In contrast, the levels of IL-1β, IL-10, GM-CSF, IFN-γ and TNF-α released into the culture medium were consistent and reproducible measured within the range of the assay (Fig 2E). When transwell inserts were tested against the T-disk for cytokine detection, the T-disk showed significantly higher release of IL-10 and IFN-γ. When analyzed in combination by two-way ANOVA test, these 5 cytokines showed a tendency towards a higher output for T-disk compared to transwell inserts, but this difference did not reach significance (Fig 2E). We did not detect a significant difference between colonic and ileac biopsies. Having established secretion of IL-1β, IL-10, GM-CSF, IFN-γ and TNF-α as robust markers in the explant assay, intra- and inter-patient variation was evaluated. Analysis of biopsies from the same site showed a low intra-patient variability, except for one patient, while inter-patient variability was much higher between patients with the same inflammation state. This is illustrated in Fig 2F with GM-CSF as a representative example, where the variability for patient 5 was the largest in the test.

To determine if cytokine release was related to the level of inflammation in the biopsy, cultured biopsies were subjected to histomorphological analysis. Biopsies were scored as non-inflamed, mildly to moderately inflamed or severely inflamed (examples in S4 Fig). The accordance between endoscopy and histopathology with regards to assessment of whether a biopsy was inflamed or non-inflamed was 81% (see S1 Table). The inflamed biopsies were almost equally distributed between mild/moderate and severe inflammation (56 and 44%, respectively).

**Fig 3:**
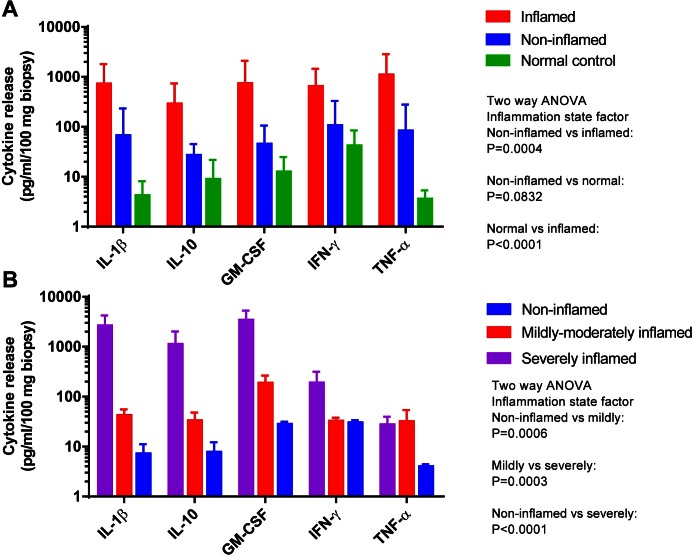
Cytokine release in conditioned medium relative to degree of inflammation as determined by endoscopy and histomorphology. Spontaneous release of the cytokines IL-1β, IL-10, GM-CSF, IFN-γ and TNF-α from (A) inflamed biopsies (n = 12 patients (2 biopsies/patient)), non-inflamed (n = 10 (2)) and normal controls (n = 6 (4)) into the culture medium measured by Bio-Plex. The inflammation states of CD biopsies were determined by the endoscopist. (B) Spontaneous release from severely inflamed (n = 3 biopsies), mildly/moderately inflamed (n = 6) and non-inflamed (n = 9) CD biopsies from 4 patients as determined by histology. Cytokine levels (pg/ml/100 mg tissue) are expressed as mean ± SEM. Statistical analyses (two-way ANOVA) were performed on the levels of the 5 cytokines in combination.

The release of IL-1β, IL-10, GM-CSF, IFN-γ and TNF-α from inflamed biopsies were found to release significantly higher levels of cytokines than both non-inflamed biopsies and biopsies from healthy controls (Fig 3). When biopsies from non-inflamed patients were compared to those from healthy controls, all 5 cytokines showed a strong trend towards increased levels for the former group, although the difference did not reach significance. Moreover, non-inflamed biopsies with histomorphology within normal limits elicited the panel of these five cytokines to a significant lower degree than both mildly-moderately and severely inflamed biopsies, while severely inflamed biopsies had a significantly increased release of the cytokines compared to mildly-moderately inflamed (Fig 3).

### Antibodies penetrate the biopsies in the explant assay

**Fig 4:**
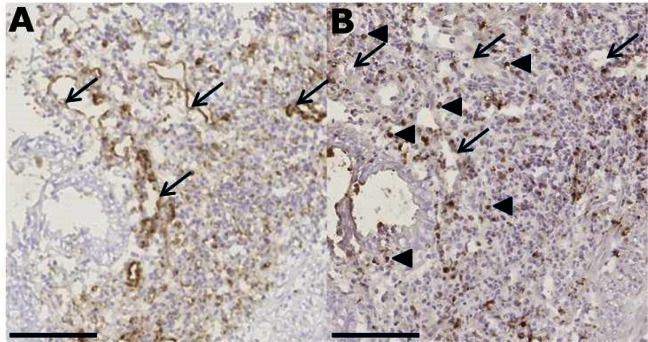
Antibodies in the culture medium diffuse into the biopsy and bind to target cells. Inflamed colonic biopsies were cultured in the presence of anti-CD31 and anti-CD3 antibodies (10 μg/ml each) for 20 hours followed by IHC staining of consecutive sections with isotype-specific anti-IgG2a (A) and anti-IgG1 (B) antibodies for detection of anti-CD31 and anti-CD3 antibodies, respectively. Arrows indicate blood vessels, which were stained in (A) by the anti-IgG2a secondary antibody specifically recognizing the anti-CD31 antibody. Blood vessels were not stained by the anti-IgG1 secondary antibody (B) that instead recognized the binding of the anti-CD3 antibody to T cells in the lamina propria (indicated by arrowheads). Bar = 100 μm.

To address the ability of the explant assay to measure responds to biological treatments, we analyzed if antibodies added to the culture medium were able to penetrate into the biopsy. Staining of consecutive sections by IHC with isotype-specific secondary antibodies against either mouse IgG1 or mouse IgG2a that recognized the anti-CD3 and the anti-CD31 antibody, respectively, showed that both antibodies could easily be detected in the same inflamed biopsy and were observed to penetrate throughout the tissue (Fig 4). The anti-CD31 antibody was detected in blood vessel-like structures consistent with binding of the antibody to endothelial cells, whereas the anti-CD3 antibody bound to scattered lymphocyte-like cells consistent with T cells. CD31-positive endothelial cells are immobile, demonstrating that test antibodies added to the culture medium indeed penetrate throughout the tissue in the explant assay.

### Cytokine release as marker for response to biological treatment

**Fig 5:**
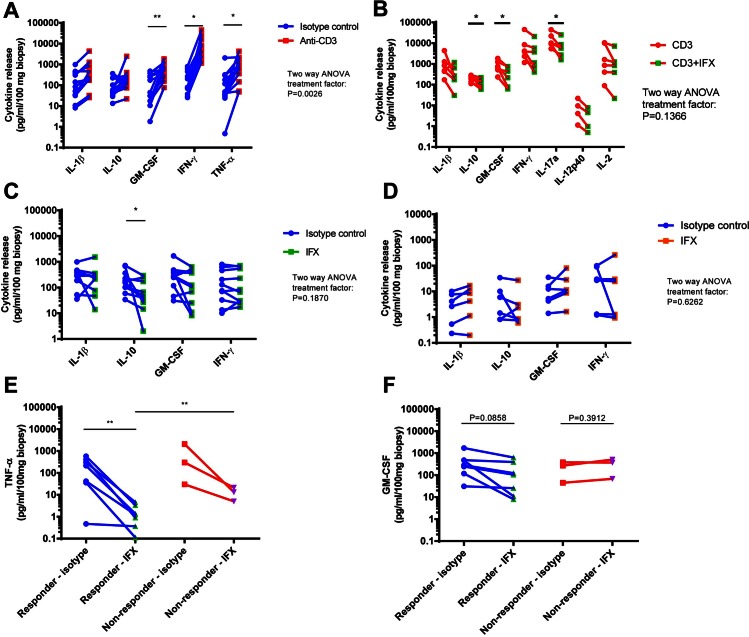
The assay responds to stimulation of the T cells and to a known biologic. (A) Cytokine release from inflamed colonic biopsies incubated with either anti-CD3 (10 μg/ml) or isotype control (10 μg/ml) in the explant assay for 24 hours (n = 12 patients, 2–4 biopsies each). Significant differences are observed for GM-CSF, IFN-γ and TNF-α (paired t-test) as well as across the panel, *P* = .0026 (two-way ANOVA). (B) Cytokine release from inflamed colonic biopsies cultured in the presence of 10 μg/ml isotype control or 10 μg/ml IFX activated by 10 μg/ml anti-CD3 for 24 hours (n = 6 patients, 4 biopsies each). IL-17a, IL-12p40 and IL-2 now reaches detection threshold under anti-CD3 stimulation. IL-10, GM-CSF and IL-17a release were significantly decreased by IFX treatment in the activated test (paired t-test). (C) Cytokine release from inflamed colonic biopsies cultured in the presence of 10 μg/ml Infliximab (IFX) or isotype control for 24 hours (n = 10 patients, 2–4 biopsies each). IL-10 release was significantly decreased by IFX treatment (paired t-test). (D) Cytokine release from healthy colonic control biopsies cultured in the presence of 10 µg/ml Infliximab (IFX) or isotype control for 24 hours (n = 6 patients, 4 biopsies each). Of the patients in (C), three were non-responders (had no effect) to IFX. All 10 are showed here divided into responders (or untested) and non-responders for TNF-αrelease (E) and GM-CSF release (F) (paired t-test within a group and un-paired t-test between responders and non-responders). *P < .05, **P < 0.01.

We finally evaluated whether a response to known biological treatments could be detected in the explant assay by measuring cytokine release into the culture medium. Anti-CD3 antibody in the medium resulted in a significant increase in release of GM-CSF, IFN-γ and TNF-α compared to cultures with isotype control (Fig 5A). IL-1β showed a strong trend towards increased release, whereas IL-10 did not change, in response to anti-CD3 treatment. When analyzed together by two-way ANOVA test, the levels of these 5 cytokines were demonstrated to be significantly increased in the culture medium from anti-CD3 treated biopsies.

When anti-TNF-α antibodies (Infliximab) were added to the culture medium together with the anti-CD3 antibody, the observed anti-CD3 induced increase in release of IL-1β, GM-CSF and IFN-γ was abrogated and replaced by a decrease, of which release of GM-CSF, but not that of IL-1β and IFN-γ, was found to be significant (Fig 5B). The anti-CD3 stimulation resulted in the cytokines IL-17a, IL12p40 and IL-2 to reach detection threshold and the effect of IFX on these cytokines were also analyzed. All three tended to be down-regulated by IFX, but only IL-17a release was significantly inhibited. Release of IL-10 was significantly reduced in response to infliximab both when added alone and in combination with anti-CD3 antibody (Fig 5C and 5B, respectively). The observed reduction of IL-10 release in response to infliximab treatment was observed with inflamed biopsies from CD patients, but not with biopsies from healthy controls. In contrast, release of IL-1β, GM-CSF and IFN-γ from neither inflamed biopsies nor biopsies from healthy controls was detected in responds to infliximab in the present study (Fig 5C and 5D, respectively). None of the patients included in this validation were currently on anti-TNF-α treatment.

TNF-α release is not shown above because of the direct impact IFX has of the cytokine, making it incomparable to the other four. When we analyze the TNF-α release by itself, we see an interesting tendency towards different detection between the group of patients who are non-responders to anti-TNF-α therapeutics and the group of responders/not tested (Fig 5E). TNF-α is neutralized or down-regulated significantly by IFX in the responder group, but not in the non-responder group. The starting concentrations are not different between the groups, but the end point is; in the responder group, the level with IFX is significantly lower. For GM-CSF (Fig 5F), the non-responders had an unchanged release (P = 0.3912), while the responders tended to have lower release as an effect of IFX (P = 0.0858).

## Discussion

Our results validate the explant assay as a test of inflammation degree and effect of biological drugs in a human experimental setting as close to clinical testing as possible. We optimized the method, showed how antibodies enter the tissue and find target cells and determined an output panel of key cytokines detecting the effect of known biologics.

Our test optimizes the previous explant assays[24, 27] by introducing the T-disk; a stable steel mesh ensuring the same volume of medium in each experiment. The reusable T-disk is more cost-efficient than transwell inserts over many tests and generally elicited higher cytokine output, maybe due to the increased contact between media and tissue compared to the transwell assays in this experiment. Histopathology and RNA analyses confirmed the excellent preservation of the biopsies over 24 hours, which is essential for reliability of the assay. The RIN score is the gold standard for evaluating the integrity of the RNA, and thus cell survival. Our result of 9.479 is considered excellent[31]. Several other studies with explant assays still submerge the tissue in media[33–35] and we have shown how disintegrated the biopsies look compared to culturing on the T-disk. IHC showed that the polarized orientation of the biopsy on the T-disk will pick up antibodies from the medium within 20 hours. The mode of entry reflecting the natural route makes it suitable for drug testing, as the antibodies found their target cells by diffusing into the tissue.

This validation of the explant assay is partly done to provide alternatives to drug development in cell-based assays and animal testing. Cell assays are preferred if the study concentrates on an effect in a specific cell type, but generally, a cell layer or even a well-constructed 3D *in vitro* model for studying IBD lack the complexity of a full and natural microenvironment present in the explant and will not reveal all intercellular pathways, especially without a valid inflammation state[17, 18, 20, 21, 36, 37]. Compared to biopsies, however, cell-based models are readily available and can deliver high-throughput screenings and specific mode-of-action studies. Animal models have the advantage of using inbred strains for experiment comparison, but it is questionable how well results can be translated into human. Here, the explant method could reduce the number of experimental animals used[16, 38]. As a model, the explant assay lacks a vascular system likely resulting in the relatively short biopsy survival. Further research should focus on extending the incubation time. Patient group sizes are heterogeneous in this study, and to confirm our findings, there is a need for larger studies with more homogenous patient populations. At this stage, the explant assay also relies on specialized equipment for analysis (multiplex) and it is relatively expensive in reagents. It should be noted that drug effects are largely influenced by elements partially or totally absent from this explant method, namely immune cell influx and microbiota. This presents as a substantial difference from *in vivo*, but it would be feasible to introduce microbiota into the explant assay as well.

Validation of the explant assay comes from showing that the delivered antibody treatments have an effect on the biopsy by quantifying the cytokine response from the tissue. Five cytokines were chosen as a panel to validate the explant assay (IL-1β, IL-10, GM-CSF, IFN-γ and TNF-α). All are key cytokines in IBD biology[10, 39, 40]. This panel was shown to be a suitable tool as markers of treatment, but not a full reflection of the pathophysiology occurring. These five cytokines are all easily detectable and have all been discussed as potential targets for IBD treatment[41–44].

The success of the method as a future standard relies on reliability of the output and subsequent ability to distinguish inflammatory states and treatments in a limited number of replications. The inter-patient heterogeneity makes it essential to perform drug efficacy tests in paired settings with biopsies from the same patient exposed to both drug and control. The intra-patient homogeneity (5 out of 6 patients) confirms the reproducibility and reliability of the explant assay, providing the option of doing testing with only a few biopsies, as biopsies are limited. The results also suggest that some biopsies taken from the same area might have different underlying inflammation states not found by the endoscopist, and that biopsies should be taken as closely as possible to counter the variability (patient 5, Fig 2F).

The trustfulness in the output enables the assay to test the biopsy inflammation state using only a few biopsies, and we find significantly higher cytokine release across the panel from inflamed biopsies compared to non-inflamed biopsies—even with the mix of non-inflamed tissue from both inflamed and uninflamed CD patients. The results also reveal how the apparent mucosal healing of the intestinal wall might elicit a different cytokine level from the inflamed state, but still tends to produce more cytokines than the tissue from normal controls not suffering from Crohn’s disease. This potentially new surrogate marker for the assessment of mucosal healing is useful since there is an increasing focus on achievement of mucosal healing in IBD, both in clinical studies and in individual patients[45, 46]. It is important to tailor the cytokine panel to the specific disease. CD has a Th1-like profile[47, 48], while ulcerative colitis has a Th2-profile[10] and irritable bowel disease has another[49]. This is mirrored in the results of the cytokine panel investigated here, reflecting the disease[50]. The levels of cytokines in this panel can predict the inflammation state mirrored in histology. The assay can clearly distinguish the three inflammation states we defined by histological evaluation and thus show the correct inflammation state of a biopsy. The assay’s ability to differentiate disease states discussed in Fig 3 comes in spite of that inherent 19% incorrect estimation by an endoscopist. The data suggest that the observed non-inflamed tissue might not be true mucosal healing, as the cytokine panel seems increased compared to healthy control. Thus, the relatively high CRP and fecal calprotectin levels in the uninflamed patients reveal that mucosal healing is difficult to determine during endoscopy or that a more proximal inflammation is present. The significance intervals comparing cytokine panel levels increase when the data is sorted into the correct inflammation states, showing high sensitivity by the assay. The explant assay’s ability to reveal the realistic inflammation state of a biopsy contrasts the inability to distinguish patients from the clinical data. The HBI score and even the fecal calprotectin and CRP analyses, normally solid diagnostic tools, could not significantly detect these inflammation differences in our population[51, 52]. It has previously been noted that histological scores could complement endoscopy scores when stratifying patients in clinical studies and the explant assay would be a valid alternative[45]. As CD is a transmural disease, the explant assay cannot alone be used as a diagnostic tool in clinical practice.

We tested the biopsies response to known treatments in the explant assay. We have validated that antibodies penetrate the biopsies and are not collected by travelling cells at the tissue surface. As expected, the T-cell stimulation by an activating anti-CD3 antibody[53] resulted in a strong increase in cytokine production across the panel. The combined panel of cytokines is significantly increased and so are the changes in GM-CSF, IFN-γ and TNF-α release specifically. This validates the explant assay as a robust detector of a treatment effect. Biopsies from the same patients were then tested with IFX together with anti-CD3. This anti-CD3 stimulation leads to detection of IL-17a, IL2 and IL12p40 as well because of the strong T cell activation[48, 54]. Infliximab binds the pro-inflammatory TNF-α from the medium and in the biopsy. Blocking TNF-α makes it impossible to distinguish the effect on TNF-α production, thus forcing us to remove TNF-α from the panel when comparing to other cytokines. The significant decrease of GM-CSF, IL-10 and IL-17a, and IL-10 only in the non-activated assay, prove that IFX modulates the tissue in the explant assay. Studies in humans suggest that reduction of the pro-inflammatory IL-17a could be an effect of IFX treatment[55, 56], and previous studies *in vitro* found that both GM-CSF[57] and IL-10[58] release were decreased by IFX, thus validating this assay as an predictor of treatment effect with a known biologic. For GM-CSF, this effect also seemed present when testing IFX responders vs non-responders. IFX does not have a detectable effect on biopsies from healthy controls. The detection of IL-12p40 in the stimulated explant assay would allow for ex vivo testing of Ustekinumab, a recent mAb therapeutic for CD[59]. The explant assay can also be stimulated with other compounds like heat-killed bacteria or LPS to suit the treatment aimed at testing.

If this assay can detect the changing levels of cytokines under IFX influence, it could also be a direct tool for distinguishing anti-TNF-α responders from non-responders when stratifying patients. Our data suggest a difference between the groups, also for GM-CSF release, but we have too few patients and this trend would be of interest to investigate further. So even though this assay detects a cytokine that an added drug neutralizes, the explant assay could detect if the concentration post-assay is low enough to be in the responder group.

The objective was to validate this assay is a simple, biologically trustworthy test system for novel therapies against IBD. The assay could also be used in other diseases with a different cytokine panel designed for testing other specific biologics, thus answering a call for improved translatable methods[15]. The explant method has already been used to investigate cytokine release and effects of new therapeutics in IBD, but before our present validation[24–28]. The assay is however unable to test drug candidates working outside the mucosa, and in CD that might be the case because of the transmural phenotype.

This explant assay holds promise to become an accepted *ex vivo* drug development platform, potentially reducing the need for cell-based assays or animal studies. Furthermore, the explant assay can be a method to screen drug effects for personalized medicine without compromising the patient or investing in a biological treatment with an unknown outcome. With over one third of CD patients being non-responders to anti-TNF-α medication, this assay could potentially test the patient for responses to the drug and help tailor a personalized treatment.

## Supporting Information

S1 Fig Morphology of porcine small intestinal tissue that have not been cultured. Non-cultured (A) compared with tissues that have cultured for 24 hours on T disk (B) or submerge in wells (C). Bar = 300 μm. HE staining. Star indicates accumulation of debris at luminal surface, arrows indicate Gruenhagen’s space and arrowheads indicate loss of mucus-containing goblet cells.
(TIF)

S2 Fig Morphology of porcine colonic tissues. Tissues that have cultured for 24 hours on T disk (A) or submerge in wells (B). Bar = 2 mm. A’ and B’ represent high magnification pictures of the area indicated by rectangles in A and B, respectively. Bar = 600 μm. HE staining.
(TIF)

S3 Fig Morphology of colonic human CD biopsies that have not been cultured. Non-cultured (A) compared with tissues that have cultured for 24 hours on T disk (B) or submerged in wells (C). Bar = 1 mm. A’, B’ and C’ represent high magnification pictures A, B and C, respectively. Bar = 100 μm. HE staining.
(TIF)

S4 Fig Histopathological evaluation of inflammation degree in biopsies subjected to explant culture. Representative pictures of H&E stained biopsies that were processed for histology after 24 hours of explant culture. Histopathological evaluation defined biopsies as non-inflamed within normal limits (A), mildly-moderately inflamed (B) and severely inflamed (C). Representative pictures are shown. Bar = 500 μm.
(TIF)

S1 File All cytokine data relating to figures. Units are pg/ml/100 mg tissue.
(XLSX)

S1 Table Histomorphological evaluation of endoscopic findings by the physician. (DOCX)
