# Supplementary figures and images for: Validation and Optimization of an Ex Vivo Assay of Intestinal Mucosal Biopsies in Crohn’s Disease: Reflects Inflammation and Drug Effects
Source: PLoS One. 2016 May 12;11(5):e0155335. doi: 10.1371/journal.pone.0155335 (PMC4865152; doi:10.1371/journal.pone.0155335)

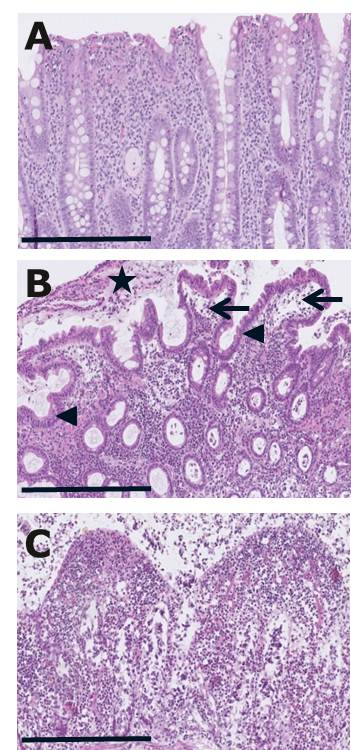

Supplement: S1 Fig — Non-cultured (A) compared with tissues that have cultured for 24 hours on T disk (B) or submerge in wells (C). Bar = 300 μm. HE staining. Star indicates accumulation of debris at luminal surface, arrows indicate Gruenhagen’s space and arrowheads indicate loss of mucus-containing goblet cells. (TIF) [file pone.0155335.s001.tif]

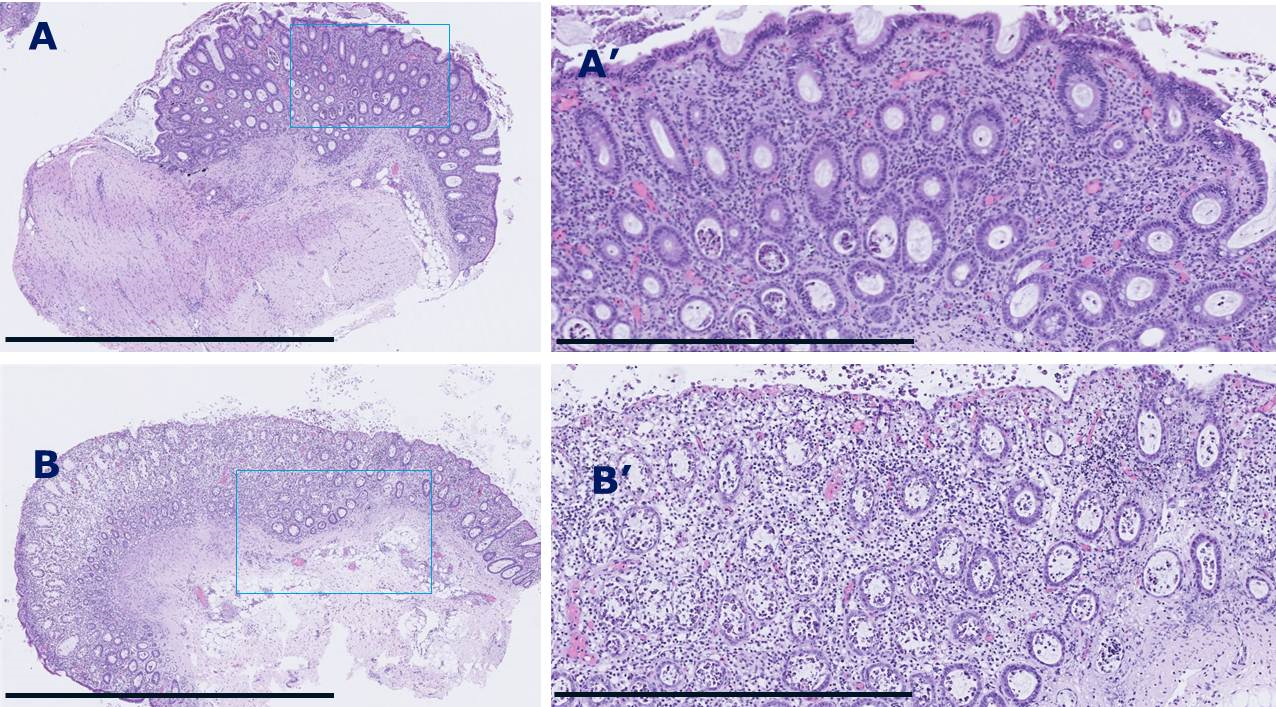

Supplement: S2 Fig — Tissues that have cultured for 24 hours on T disk (A) or submerge in wells (B). Bar = 2 mm. A’ and B’ represent high magnification pictures of the area indicated by rectangles in A and B, respectively. Bar = 600 μm. HE staining. (TIF) [file pone.0155335.s002.tif]

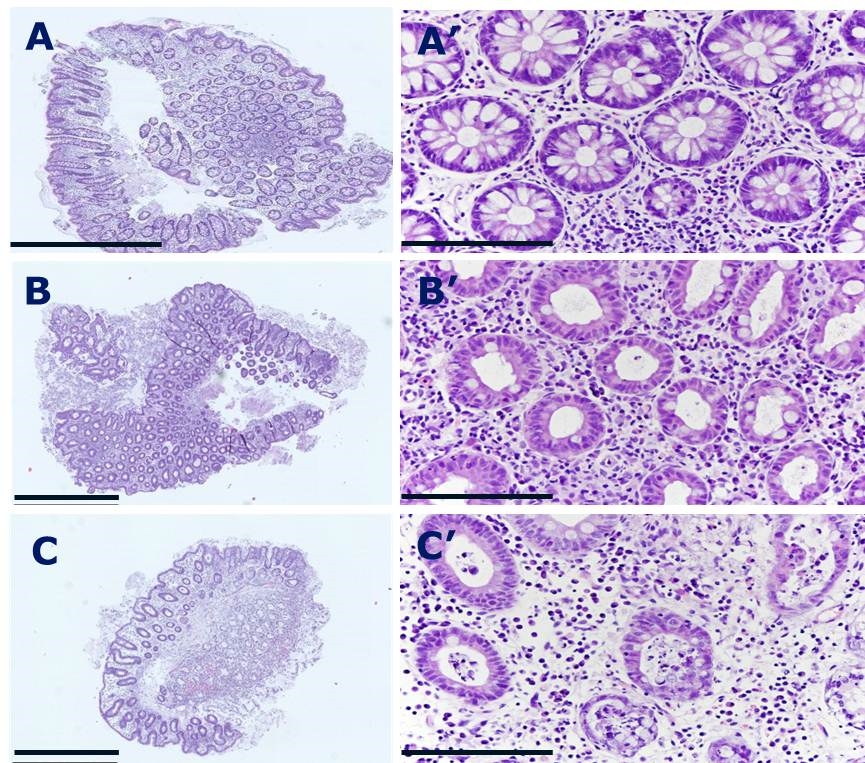

Supplement: S3 Fig — Non-cultured (A) compared with tissues that have cultured for 24 hours on T disk (B) or submerged in wells (C). Bar = 1 mm. A’, B’ and C’ represent high magnification pictures A, B and C, respectively. Bar = 100 μm. HE staining. (TIF) [file pone.0155335.s003.tif]

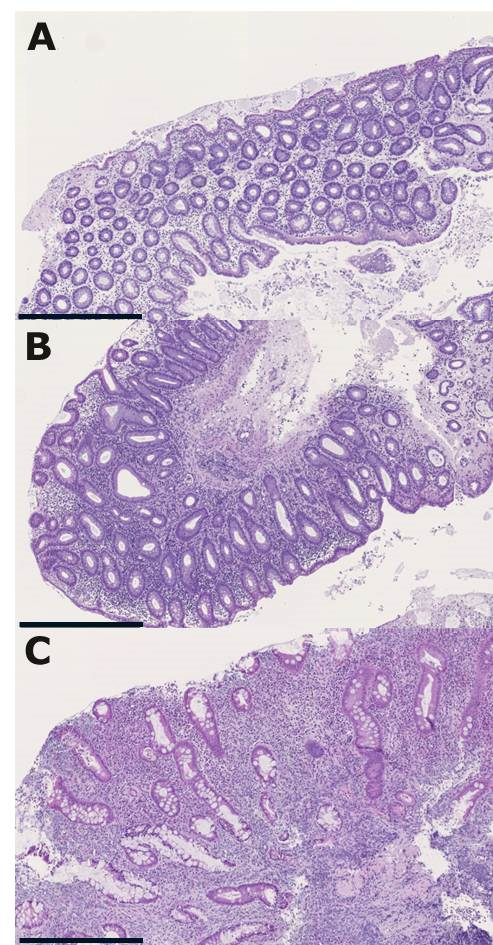

Supplement: S4 Fig — Representative pictures of H&E stained biopsies that were processed for histology after 24 hours of explant culture. Histopathological evaluation defined biopsies as non-inflamed within normal limits (A), mildly-moderately inflamed (B) and severely inflamed (C). Representative pictures are shown. Bar = 500 μm. (TIF) [file pone.0155335.s004.tif]
