# Supplementary material for: Validation and Optimization of an Ex Vivo Assay of Intestinal Mucosal Biopsies in Crohn’s Disease: Reflects Inflammation and Drug Effects
Source: PLoS One. 2016 May 12;11(5):e0155335. doi: 10.1371/journal.pone.0155335 (PMC4865152; doi:10.1371/journal.pone.0155335)
Supplement: S1 Table — (DOCX) [file pone.0155335.s006.docx]

| Assessment of biopsy inflammation state during endoscopy (n=32) | Histomorphological assessment: | |  |  |
| --- | --- | --- | --- | --- |
|  | Non-inflamed | Mild/moderate inflammation | Severe inflammation | Correct assessed by histology |
| Non-inflamed | 13 | 3 | 0 |  |
| Inflamed | 3 | 6 | 7 |  |
| Overall |  |  |  | 81.25% |

NOTE. Blinded evaluation performed by two investigators.
